# The Cobalamin-Binding Protein in Zebrafish Is an Intermediate between the Three Cobalamin-Binding Proteins in Human

**DOI:** 10.1371/journal.pone.0035660

**Published:** 2012-04-20

**Authors:** Eva Greibe, Sergey Fedosov, Ebba Nexo

**Affiliations:** 1 Department of Clinical Biochemistry, Aarhus University Hospital, Aarhus, Denmark; 2 Department of Molecular Biology and Genetics – Department of Molecular Biology, Aarhus University, Aarhus, Denmark; Consejo Superior de Investigaciones Cientificas, Spain

## Abstract

In humans, three soluble extracellular cobalamin-binding proteins; transcobalamin (TC), intrinsic factor (IF), and haptocorrin (HC), are involved in the uptake and transport of cobalamin. In this study, we investigate a cobalamin-binding protein from zebrafish (*Danio rerio*) and summarize current knowledge concerning the phylogenetic evolution of kindred proteins. We identified a cobalamin binding capacity in zebrafish protein extracts (8.2 pmol/fish) and ambient water (13.5 pmol/fish) associated with a single protein. The protein showed resistance toward degradation by trypsin and chymotrypsin (like human IF, but unlike human HC and TC). The cobalamin analogue, cobinamide, bound weaker to the zebrafish cobalamin binder than to human HC, but stronger than to human TC and IF. Affinity for another analogue, adenosyl-pseudo-cobalamin was low compared with human HC and TC, but high compared with human IF. The absorbance spectrum of the purified protein in complex with hydroxo-cobalamin resembled those of human HC and IF, but not TC. We searched available databases to further explore the phylogenies of the three cobalamin-binding proteins in higher vertebrates. Apparently, TC-like proteins are the oldest evolutionary derivatives followed by IF and HC (the latter being present only in reptiles and most but not all mammals). Our findings suggest that the only cobalamin-binding protein in zebrafish is an intermediate between the three human cobalamin binders. These findings support the hypothesis about a common ancestral gene for all cobalamin-binding proteins in higher vertebrates.

## Introduction

In humans, the uptake and transport of cobalamin (vitamin B_12_) is mediated by three structural kindred proteins: transcobalamin (TC) that transports cobalamin through the bloodstream and ensures its cellular uptake; haptocorrin (HC) that carries most of the circulating cobalamin as well as inactive analogues of the vitamin; and intrinsic factor (IF) that facilitates the intestinal uptake of the vitamin [1], [2]. Despite the various locations and functions, all three proteins have a polypeptide backbone of similar size [3] with a highly conserved region (the so-called cobalamin-binding signature) [4]. Yet, the overall homology of the proteins is only 25–30% [5]. The cobalamin-binding proteins seemingly evolved from a common ancestral gene [3], [4], [6], where sequential duplication of genes caused divergence of IF from TC and later on HC from IF [1]. While human IF and HC are glycoproteins and their genes are localized in chromosome 11 [7], [8], human TC is non-glycosylated and its gene is localized in chromosome 22 [9].

The phylogenetic search for cobalamin binders within the subphylum vertebrata has mostly been restricted to mammals. The proteins TC and IF (as well as their encoding genes, *TCN2* and *GIF*, respectively) have been found in all investigated species, including mouse, rat, hog, cow, and chimpanzee [10]. HC and/or its encoding gene, *TCN1*, have been found in many mammals including hog [6], rabbit [11], and cow [12]. Yet, recent studies show that mice lack HC, and that TC is their only cobalamin transporter in blood resembling both human TC and HC [13].

Zebrafish (*Danio rerio*) is an important vertebrate model organism, which has an advantage of rapid embryonic development if compared with higher vertebrate models, such as rats and mice. As mammals and other vertebrates, the zebrafish have two intracellular cobalamin-dependent enzymes, methylmalonyl coenzyme A mutase (NCBI GeneID 569581) and 5-methyl tetrahydrofolate homocysteine methyl transferase (NCBI GeneID 378847) [10], which share an overall amino acid identity with the corresponding human enzymes of 81% and 78%, respectively. These two cobalamin-dependent enzymes are necessary for growth and reproduction [2]. Zebrafish are omnivorous and feeds on zooplankton, insects and insect larvae [14] for source of cobalamin.

In this study, we searched for soluble cobalamin-binding proteins in zebrafish and found only one resembling an intermediate between the cobalamin-binding proteins found in humans. Analysis of the sequence databases indicated that the cobalamin-binding proteins in higher vertebrates might have descended from a common ancestral gene *after* divergence of the bony fish (Osteichthyes).

## Materials and Methods

### Protein Extractions

Zebrafish were kept in tap water with 0.02% Aquarium START Plus Solution (DajanaPet, Bohunovice, Czech Republic) and were fed Flakes Complete Fish food (BestFriend, Kuopio, Finland) according to the manufactures' instructions. On the day of sacrifice, zebrafish were homogenized on ice in 10 mM PIPES pH 7.4 (Sigma-Aldrich, Broendby, Denmark), 1 mM EDTA (Sigma-Aldrich, Broendby, Denmark), 3 mM MgCl_2_, 6H_2_O (Merck, Damstadt, Germany), 400 mM NaCl, and two tablets per 50 ml buffer of protease inhibitor cocktail (Cat. No. 11697498001, Roche Diagnostics, Mannheim, Germany) using a tissue rupture (Qiagen, Copenhagen, Denmark). For studies investigating the effect of trypsin and chymotrypsin on protein stability, no protease inhibitors were added to the buffer before homogenization. To ensure cell rupture, the homogenates were subjected to three freeze-thaw cycles followed by three times 10 seconds of ultra sonication (MSE probe universal). Finally, protein extracts were centrifuged at 4°C for 40 minutes at 20.000×g, and the supernatants were stored at −20°C until analysis. According to the "European convention for the protection of vertebrate animals used for experimental and other scientific purpose" no approval is necessary for the humane sacrifice of the zebrafish used in this study.

Human spermal fluid and gastric juice were collected by medical staff as part of diagnostic tests for acidity (gastric juice, Bispebjerg Hospital, Copenhagen, Denmark) and infertility (spermal fluid, Hillerod Hospital, Hillerod, Denmark) [15]. Excess samples were collected with no information's allowing the samples to be traced back to the donor. Human saliva was collected as part of a research project on HC in saliva previously published [16] and as stated in the paper all of the individuals gave informed consent.

### Unsaturated Cobalamin Binding Capacity and Total Cobalamins

Unsaturated cobalamin binding capacity (UB_12_BC) was measured as described by Gottlieb *et al* [17]. In brief, 100 µl sample material was incubated with 25 µl radiolabeled cobalamin (^57^[Co]-cobalamin) (5 nM) (Kem-En-Tec, Taastrup, Denmark) in 200 µl 0.1% phosphate-buffered bovine albumin (PBA) for 15 minutes, and 500 µl hemoglobin-coated charcoal solution was added to precipitate unbound cobalamin. The charcoal solution was prepared by mixing equal volumes of a 5% aqueous suspension of activated charcoal (Sigma-Aldrich, Broendby, Denmark) with a 0.5% aqueous solution of bovine hemoglobin (Becton Dickinson, Broendby, Denmark). After additional 10 minutes of incubation, charcoal-bound cobalamin was precipitated by centrifugation for 10 minutes at 2600×g. Finally, the supernatants were measured for radioactivity in the Wizard Automatic Gamma Counter (Perkin Elmer).

Total cobalamins were measured using Cobas 6000 E immunoassay system (Roche Diagnostics, Hvidovre, Denmark) with a detection range of 55–1476 pM. Zebrafish protein extracts were diluted five times with 0.9% NaCl solution prior to analysis.

### Collection of Secreted Cobalamin-binding Protein from the Ambient Water

Five zebrafish were kept in 100 ml tap water for six hours to allow secretion of cobalamin-binding protein into the medium. The water was collected and stored at −20°C until further analysis. For each collection, 1 ml water sample was taken out before storage, and sample solvents were evaporated in a Hetovac vacuum centrifuge (HETO, Allerod, Denmark). Pellets were resuspended in 100 µl NaH_2_PO_4_ pH 7.5 and subjected to measurement of unsaturated cobalamin binding capacity.

### Size Exclusion Chromatography

For size exclusion chromatography, 100 µl zebrafish protein extract (∼1800 pmol/L UB_12_BC) or 100 µl collected zebrafish-ambient-water (∼ 700 pmol/L UB_12_BC) were incubated with 50 µl ^57^[Co]-cobalamin (5 nM) for 30 minutes at room temperature. Then, the samples were applied to a Superdex 200 column (GE Healthcare, Broendby, Denmark) attached to a Dionex ICS-3000 HPLC system. Collected fractions were measured for radioactivity in the Wizard Automatic Gamma Counter and for cobalamin content in the Cobas 6000 E immunoassay system. Blue Dextran (Sigma-Aldrich, Broendby, Denmark) and Na^22^ (GE Healthcare, Broendby, Denmark) was used for determination of void volume (V_0_) and total volume (V_t_), respectively. For comparison, size exclusion chromatography of the human cobalamin-binding proteins, TC, HC, and IF, was performed.

### Degradation with Trypsin and Chymotrypsin

The sensitivity of the cobalamin-binding protein from zebrafish toward enzymatic cleavage was explored using increasing concentrations of trypsin and chymotrypsin. For each protein extract (zebrafish cobalamin binder, human TC, HC, and IF), the UB_12_BC was adjusted to 1 nM by dilution with 0.1 M Tris-HCl pH 7.4. Then, 0.4 pmol apo-protein (400 µl) was incubated with 25 µl ^57^[Co]-cobalamin (5 nM) in 0.1 M Tris-HCl pH 7.4 in a total volume of 800 µl for 30 minutes at room temperature. Increasing concentrations (0 units to 500 units) of trypsin (Sigma, Broendby, Denmark) or chymotrypsin (Sigma, Broendby, Denmark) in a total volume of 100 µl 0.1 M Tris-HCl pH 7.4 were added, and the mixture was incubated for 18 hours at 37°C. Unbound cobalamin was absorbed on hemoglobin-coated charcoal (500 µl, 10 minutes) and precipitated by centrifugation for 10 minutes at 2600×g. The radioactivity of supernatants was measured for radioactivity in a Wizard Automatic Gamma Counter.

### Binding to Concanavalin A

The presence of carbohydrates in the cobalamin-binding protein from zebrafish was examined by precipitation with Con A Sepharose (Amersham Bioscience, Uppsala, Sweden), a lectin known to bind α-D-mannopyranosyl, α-D-glucopyranosyl, and kindred compounds. For each protein extract (zebrafish cobalamin binder, human TC, HC, and IF), the UB_12_BC was adjusted to 2 nM by dilution with binding buffer (20 mM Tris-HCl, pH 7.4, 0.5 M NaCl), and 600 µl of each sample was incubated with 10 µl ^57^[Co]-cobalamin (5 nM) for 30 minutes at room temperature. A 100 µl suspension of Con A Sepharose (prepared accordingly to the manufacturer's instructions) was mixed with 200 µl of the binding buffer, and the samples were incubated for 1 hour at room temperature before centrifugation (5 minutes at 8000×g). The ^57^[Co]-cobalamin present in the supernatant and precipitate was measured in a Wizard Automatic Gamma Counter.

### Corrinoid Binding Assay

The ability of zebrafish extract to bind different corrinoids was explored using a competitive assay [18]. The ligands in question were: cyano-cobalamin (Sigma-Aldrich, Broendby, Denmark), dicyano-cobinamide (Sigma-Aldrich, Broendby, Denmark) and adenosyl-pseudo-cobalamin (synthesized and described earlier [19]). The three corrinoids were diluted with 0.1% PBA to concentrations ranging from 0 nM to 355 nM, and mixed with 50 µl ^57^[Co]-cobalamin (0.4 nM) tracer solution prior to addition of protein extract (UB_12_BC adjusted to 0.3 nM). The mixture was incubated for 18 hours at 4°C, whereupon the unbound cobalamin was precipitated, and the radioactivity of supernatant was measured in a Wizard Automatic Gamma Counter. For a comparison, the human cobalamin-binding proteins, TC, HC, and IF, were also examined.

### Purification of Zebrafish Cobalamin Binding Protein

Unsaturated cobalamin-binding protein secreted from the zebrafish into the ambient water was purified on an affinity column with hydroxy-cobalamin coupled to a sepharose matrix [20]. In brief, EAH Sepharose 4B (GE Healthcare, Uppsala, Sweden) was equilibrated with hydroxy-cobalamin (1 mg/ml) (GEA, Copenhagen, Denmark) in 0.1 M NaH_2_PO_4_ pH 7.5 and incubated at 57 °C for five hours, while gently mixing the solution every 20–30 minutes. Sodium azide was added to a final concentration of 0.02%, and the suspension was placed at 4°C for 20 hours to stabilize the thermo-labile bond between the cobalt atom of cobalamin and the amino group of the sepharose. Prior to use, the cobalamin-coupled sepharose was extensively washed with 50 volumes of cold 0.1 M NaH_2_PO_4_ pH 7.5 followed by 30 volumes of demineralized water and six volumes of 0.1 M NaH_2_PO_4_ pH 7.5 to eliminate the excess of free cobalamin. The collected zebrafish-ambient-water (100 ml) was applied to the column with a flow rate that guaranteed five minutes residence time of the solution inside the matrix. Afterwards, the column was washed with 15 volumes of 0.1 M Tris pH 7.5 with 1 M NaCl and with three volumes of 0.1 M NaH_2_PO_4_ pH 7.5. Absorbed cobalamin-binding protein was detached from the column after incubation at 37°C for 24 hours, and it was eluted with two volumes of 0.1 M NaH_2_PO_4_ pH 7.5 warmed to 37°C. The eluate was subjected to six hours dialysis in 14 kDa cut-off dialysis tubes (Medicell International, London, England) against 0.1 M NaH_2_PO_4_ pH 7.5 followed by 18 hours dialysis against demineralized water. Finally, the solvents were evaporated in a Hetovac vacuum centrifuge, and the pellet was resuspended in 0.1 M NaH_2_PO_4_ pH 7.5. Recovery was judged by comparison of the collected cobalamin with the amount of UB_12_BC loaded into the column.

Purity of the protein sample was evaluated by 12% SDS-PAGE, adding 25 µl (12 µg) of the protein per lane of a precast Tris-HCl gel (Bio Rad, Hercules, California, USA) stained by Coomassie Brilliant Blue according to the standard procedure.

### Absorbance Spectrum

The absorbance spectrum of the purified zebrafish cobalamin binder in complex with hydroxo-cobalamin was recorded on a Varian Cary 50 spectrophotometer (Varian A/S, Sydney, Australia). Spectral transition upon addition of exogenous 15 mM histidine was examined as a test on the coordination of the endogenous His-residue to the cobalt ion [21].

### In Situ Sequence Analyses

The National Center for Biotechnology Information (NCBI) database [10] and the University of California Santa Cruz (UCSC) Genome Browser [22] were searched for TC, HC, and IF nucleotide sequences in registered vertebrates using the search terms “TCN1”, “TCN2”, and “GIF”. Protein sequence alignments were performed using the ClustalW Alignment method at the homepage of the European Molecular Biology Laboratory-European Bioinformatics Institute (EMBL-EBI) [23] using the default settings. Sequences with less than 20% of overall identity to the human cobalamin binders were excluded. Conserved regions, including the cobalamin binding site, were identified using the NCBI BLAST tool [24].

## Results

### Total Cobalamins and Unsaturated Cobalamin Binding Capacity in Zebrafish

**Figure 1:**
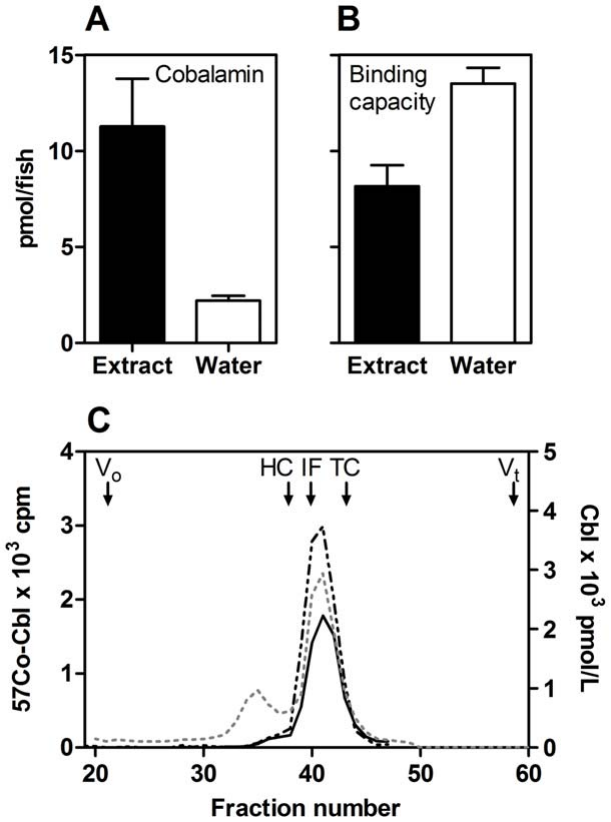
Secretion of Zebrafish Cobalamin-binding Protein to the Ambient Water. Five zebrafish were kept in 100 ml tapped water for six hours and content of cobalamin (A) and unsaturated cobalamin binding capacity (B) in the zebrafish ambient water was measured and compared to the values found in zebrafish protein extracts. Mean and SEM of three independent studies are indicated. (C) Size exclusion chromatography of the cobalamin binders found in zebrafish protein extract (full drawn line) and in collected zebrafish-ambient-water (black dashed line) measured as unsaturated cobalamin binding capacity, ^57^[Co]-cobalamin (57Co-Cbl) (left Y-axis), and as amount of protein-bound cobalamin (Cbl) in the protein extract (grey dashed line) (right Y-axis). X-axis indicates the fraction number. Elution volume for void volume (V_0_), human HC, IF, TC, and total volume (V_t_) are shown by arrows.

Protein extracts of homogenized zebrafish (n = 5) contained 11.3 [6.3–14.2] pmol cobalamin/fish (mean and [range]) and had an unsaturated cobalamin binding capacity of 8.2 [6.3–10.1] pmol/fish (Figure 1). Provided that all cobalamin is protein bound, one zebrafish is anticipated to contain 19.5 [12.6–24.3] pmol of a cobalamin-binding protein.

### Secretion of Cobalamin-binding Protein to the Ambient Water

Five zebrafish were kept in 100 ml tap water for six hours before the concentration of cobalamin and the unsaturated cobalamin binding capacity of the ambient water were determined. The water was found to contain 112 [98–135] pmol/l cobalamin (or 2.2 [1.9–2.7] pmol cobalamin/fish) (Figure 1A). Also, a significant unsaturated cobalamin binding capacity of 677 [620–756] pmol/l (or 13.5 [12.4–15.1] pmol/fish) was detected in the ambient water (Figure 1B), whereas none was found in the tap water (before addition of fish), fish food (Flakes Complete Fish food), or Aquarium START Plus Solution (used in aquarium for protection of the fish skin mucus) (data not shown). A low binding capacity (1.1 [0.81–1.35] pmol/fish) was found in fish feces (data not shown). Employing size exclusion chromatography, the cobalamin unsaturated binding protein from zebrafish protein extracts and zebrafish ambient water displayed similar gel filtration patterns (Figure 1C). In zebrafish protein extract, we found cobalamin to elute with a major peak together with the unsaturated cobalamin-binding proteins and in addition we observed a minor peak with a larger molecular size, possibly representing cobalamin bound to the intracellular cobalamin-dependent enzymes.

### Proteolytic Sensitivity of Zebrafish Cobalamin-binding Protein

**Figure 2:**
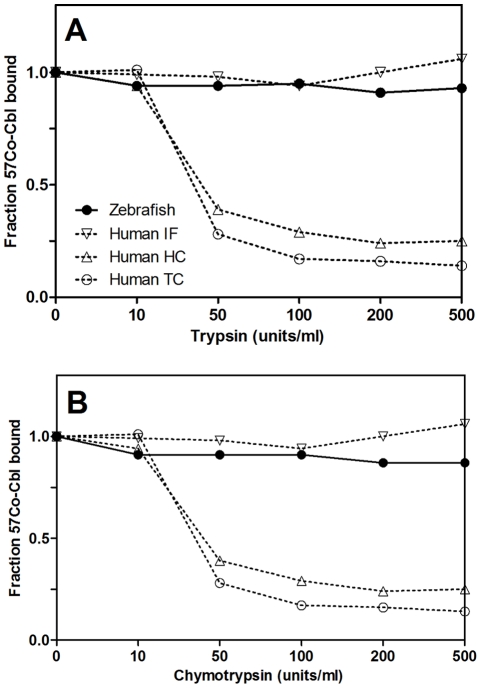
Sensitivity of the Zebrafish Cobalamin Binder toward Degradation by Trypsin and Chymotrypsin. The cobalamin binder from zebrafish and human TC, HC, and IF were incubated with ^57^[Co]-cobalamin for 30 minutes before addition of increasing concentrations of trypsin and chymotrypsin (incubation for 18 hours at 37°C). Protein-bound ^57^[Co]-cobalamin was measured after removal of free cobalamin by charcoal precipitation, and the amount of ^57^[Co]-cobalamin bound was expressed relative to the amount bound when only ^57^[Co]-cobalamin was present.

We investigated sensitivity of the zebrafish cobalamin binder, as compared to the human cobalamin binders, toward proteolytic degradation caused by addition of increasing concentrations of trypsin and chymotrypsin. While human TC and HC were degraded by trypsin and chymotrypsin; the zebrafish cobalamin binder and human IF remained intact under the experimental conditions (Figure 2).

The enzymatic resistance of the cobalamin-binding protein from zebrafish was not caused by a carbohydrate coating, since the protein did not interact with Con A (data not shown).

### Binding of Corrinoids to the Zebrafish Cobalamin Binder

Human TC, HC, and IF have comparable high affinities for the physiological active forms of cobalamin (K_d_<<1 pM)[21], but exhibit different selectivity toward non-functional cobalamin analogues [21]. Since only HC binds cobinamide under physiological conditions, and because only HC and TC interact reasonably well with adenosyl-pseudo-cobalamin [21]; these corrinoids were used to test relation of the zebrafish cobalamin binder to one or another type of the human binders.

**Figure 3:**
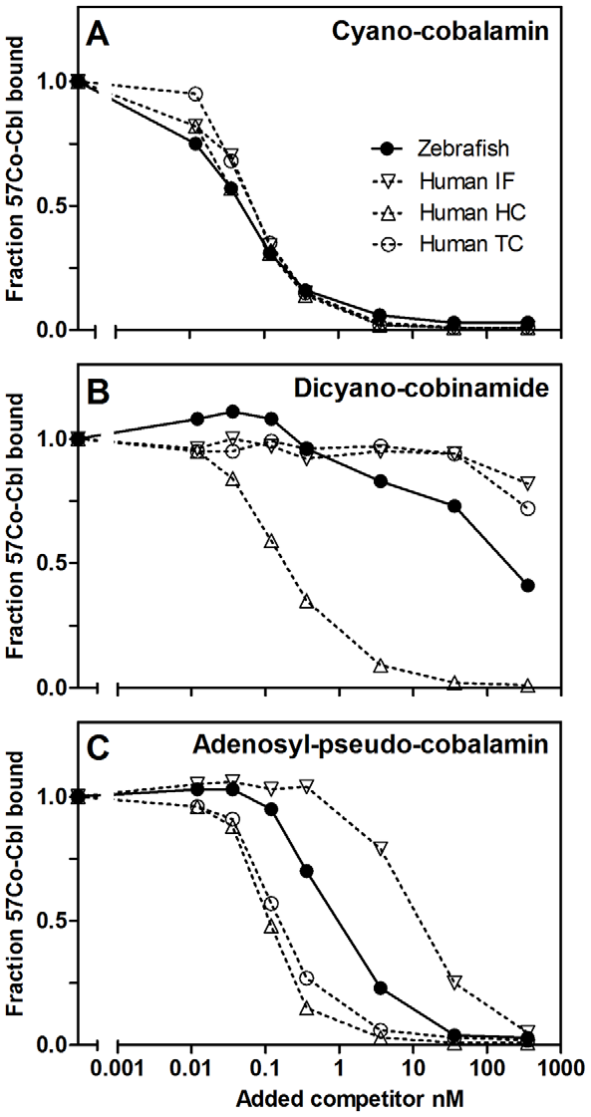
Ligand Binding Characteristics of the Cobalamin Binder from Zebrafish. The zebrafish cobalamin binder (as well as human TC, HC, and IF) was incubated for 18 hours with ^57^[Co]-cobalamin and increasing concentrations of the unlabeled ligand: cyano-cobalamin (A), dicyano-cobinamide (B) and adenosyl-pseudo-cobalamin (C). Protein-bound ^57^[Co]-cobalamin was measured after removal of free cobalamin by charcoal precipitation, and the amount of protein-associated ^57^[Co]-cobalamin was expressed relative to the amount bound when only ^57^[Co]-cobalamin was present.

The zebrafish protein displayed cobalamin-binding curves comparable to those of human TC, HC, and IF (Figure 3A), suggesting an equivalent affinity toward cobalamin. On the contrary, the binding curves for cobinamide (Figure 3B) and adenosyl-pseudo-cobalamin (Figure 3C) demonstrated a unique pattern, which distinguished the zebrafish protein from any of the human binders. The affinity of the zebrafish cobalamin binder toward cobinamide was lower than the affinity of HC, but higher than the affinity of TC and IF (Figure 3B), and the affinity toward adenosyl-pseudo-cobalamin was lower than the affinity of TC and HC, but higher than the affinity of IF (Figure 3C).

### Purification of Zebrafish Cobalamin-binding Protein and Spectral Studies

**Figure 4:**
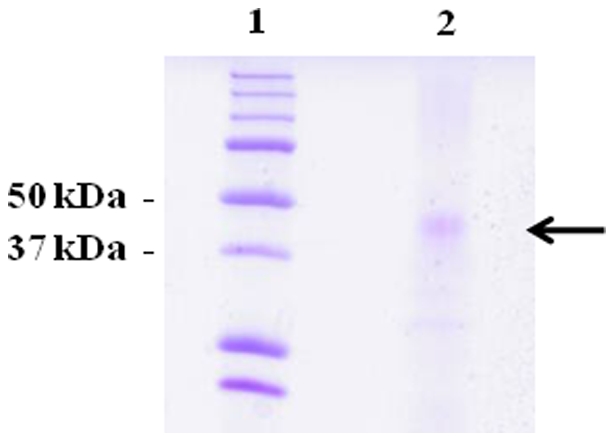
Gel Electrophoresis of Purified Zebrafish Cobalamin Binder. The cobalamin-binding protein purified from zebrafish is shown as a major band with an apparent molecular mass of approximately 40 kDa. Lane 1) marker; Lane 2) purified zebrafish cobalamin binder (see Materials and Methods for details).

We purified the cobalamin binder secreted by zebrafish into the ambient water using affinity chromatography on the collected water (100 ml, UB_12_BC ∼700 pmol/L). This source was chosen because a lower amount of contaminants was present in comparison to the protein extract (not shown). As judged by the reduced SDS-PAGE, the purified protein had an approximate molecular mass of 46 kDa (Figure 4), which is similar to the molecular mass of human TC [5].

**Figure 5:**
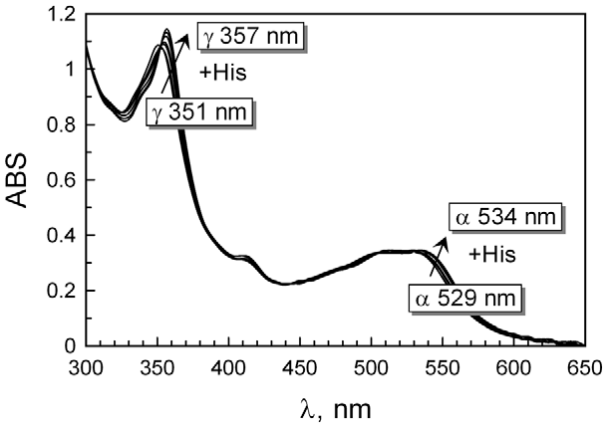
Absorbance Spectra of Cobalamin bound to Purified Zebrafish Cobalamin Binder. Spectral characteristics of the zebrafish cobalamin binder in complex with hydroxo-cobalamin are shown. Spectral transition after addition of 15 mM histidine was recorded after 1, 3, 5, 12, 20, and 30 minutes of incubation at room temperature. The arrows indicate the direction of this transition.

The absorbance spectrum of the purified cobalamin binder from zebrafish in complex with hydroxo-cobalamin (≈ 40 µM) was examined and compared to those of human TC, HC, and IF, described earlier [25]. The record scan (Figure 5) displayed the γ-peak at 351 nm and the α-peak at 529 nm showing a resemblance to the analogous spectra of human HC (γ = 356 nm, α = 528 nm) and IF (γ = 356 nm, α = 531 nm), but differing from the TC spectrum (γ = 362 nm, α = 546 nm) [25]. Addition of 15 mM His to the examined protein-cobalamin complex resulted in a spectral shift that developed over time (τ½ ≈ 5 minutes). This effect indicates coordination of the exogenous His to cobalt ion of cobalamin, which is possibly only if the upper OH_2_-group of hydroxo-cobalamin is unprotected (feature of human HC and IF). Similar spectral changes were observed earlier in human HC and IF but not in human and bovine TCs, where the endogenous His-residue of the binder is already coordinated to cobalt ion and hinders interaction with the exogenous His [25].

### In Situ Sequence Analyses

**Figure 6:**
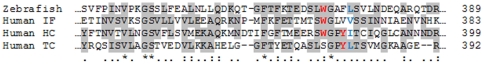
Partial Protein Sequence Alignment of the Primary Cobalamin Binding Site in the C-terminus. The sequence alignment of C-terminal domains of the zebrafish cobalamin binder and the human cobalamin binders was done in ClustalW2 using default settings. The residues highlighted in red residues are the ones that form hydrophobic interactions with the ligand and that are characteristic for the specificity for cobalamin (TC, HC, IF) and other corrinoids (only HC) [26]. The residues highlighted in blue form hydrogen bonds to the ligand [26]. Residue identities are on a gray background. The numbers in the right margin refers to the specific amino acids in the full-length protein including signal peptides.

According to the NCBI database [10], the zebrafish genome contains two soluble cobalamin-binding proteins; one predicted to be a HC-like (NCBI GeneID 566714) and one predicted to be a TC-like type (NCBI GeneID 407646). However, the HC-resembling sequence (NCBI GeneID 566714) (142 aa) encoded a protein, which was considerably smaller than all the cobalamin binders described so far [3]. In addition, its overall amino acid identity to the human variants was quite low: 9.2%. 9.0%, and 9.2% for HC, TC, and IF, respectively. The sequence contained neither conserved domains nor the cobalamin-binding site, and therefore, it was excluded from the further analysis. The TC-like sequence (NCBI GeneID 407646) (429 aa) showed a higher overall identity to TC (30.2%) than HC (25.4%) and IF (26.4%). Yet, its primary cobalamin-binding site at C-terminus showed a structural resemblance to IF when looking at the combination of conserved residues important for ligand specificity for cobalamin and other corrinoids (Figure 6, highlighted in red). The zebrafish cobalamin-binding site contained a Trp^372^-residue (Trp^366^ in IF, Trp^382^ in HC) not found in TC [26], and the presence of this Trp-residue but lack of a Tyr-residue (Tyr^385^ in HC, Tyr^380^ in TC) is a characteristic trait of IF [26] (Figure 6).

### In Situ Phylogenic Studies

**Figure 7:**
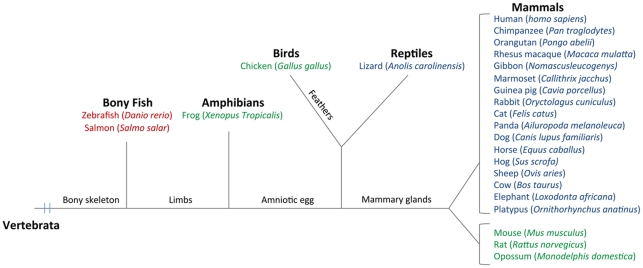
Phylogenetic Relationship of the Cobalamin Binding Proteins TC, HC, and IF. The phylogenetic tree is constructed from nucleotide search using the NCBI- and UCSC genome browser websites in registered vertebrates. Species are colored recording to the findings of one (predicted to be TC-like) (red), two (TC and IF) (green), or three (TC, IF, and HC) (blue) cobalamin binders in the databases. The accessions numbers for the sequences are listed in the supplementary data (Table S1).

The occurrence of the cobalamin-binding proteins, TC, HC, and IF, in different species within the subphylum vertebrata was investigated using NCBI and UCSC search tools [10], [22], and the results are depicted in Figure 7. Sequences with an overall identity to the human cobalamin binders below 20% were not included in the study. TC and IF were found in all investigated mammals, as well as in birds (chicken), reptiles (lizard), and amphibians (frog). HC was also found in most mammals, but not in rodents (mouse, rats) or marsupials (opossum). HC was found in reptiles (lizard), but not in birds or amphibians. In bony fish (zebrafish and salmon), we only found one cobalamin-binding protein; in both cases predicted to be a TC-like type based on the sequence. The accessions numbers for all sequences used in Figure 7 are listed in the supplementary data (Table S1).

## Discussion

In the present study, we investigated the soluble cobalamin-binding protein from zebrafish (*Danio rerio*) and compared it with the three known soluble extracellular cobalamin-binding proteins present in humans and other mammals. Those are IF, the protein facilitating intestinal uptake of cobalamin; TC, the protein mediating the uptake of cobalamin by the cells of the body; and HC, a protein of unknown function present in plasma and most extracellular fluids [1], [2]. We report that zebrafish contained only one cobalamin-binding protein that resembled an intermediate form of the cobalamin binders found in humans. Consequently, the suggested name for this protein could be an abbreviation of HC, IF, and TC: zebrafish HIT protein.

We found that the zebrafish excreted large amounts of the unsaturated cobalamin-binding protein to the ambient water resembling the secretion of HC into most extracellular fluids in mammals. Recently, zebrafish were found to secrete other proteins, such as trypsin, from gill epithelial cells to the surroundings in response to stress when being transferred to distilled water [27]. Likewise, the secretion of cobalamin-binding protein could be induced by stress; however the physiological role of this secretion remains to be clarified.

Based on the studies of raw protein extract, we found that the cobalamin binder from zebrafish is resistant toward degradation by trypsin and chymotrypsin (like IF). Yet, it did not bind to Con A Sepharose and seemingly lacks carbohydrates (like TC). In addition, the zebrafish protein displayed intermediate affinities for the cobalamin analogues cobinamide and adenosyl-pseudo-cobalamin, which was not consistent with any of the human type proteins (HC, TC, and IF).

In order to further characterize the protein, we purified it by affinity chromatography. The characterization covered only a few basic features, because of a limited amount of available material (all together, we purified 1.3 nmol of the protein). The zebrafish cobalamin binder had a molecular mass of approximately 46 kDa, which is similar to human TC and expected from the amino acid sequence (429 aa) found in the NCBI database (GeneID 407646). In contrast, the spectral properties of the zebrafish binder resembled those of HC and IF. Absence of protection of the cobalt ion in bound hydroxo-cobalamin clearly distanced the zebrafish protein from human and bovine TCs (both exhibiting this protection).

In summary, we found the cobalamin-binding protein from zebrafish to be a structural hybrid between the human cobalamin-binding proteins. Based on the sequence collected from the databases, the zebrafish cobalamin binder has the highest overall identity with TC, but the amino acid composition in the cobalamin-binding site resembles mostly IF.

These findings in zebrafish suggest that the three known cobalamin-binding proteins in higher vertebrates have descended from a common ancestral gene *after* divergence of the bony fish (Osteichthyes). This is supported by the in situ phylogenetic studies presented in Figure 7, where only one cobalamin binder is found in bony fish; two cobalamin binders, IF and TC, are found in lower vertebrates, and all three cobalamin binders are found in higher vertebrates. This scheme correlates with the hypothesis that HC has evolved from duplication of the IF gene, which in its turn has evolved from duplication of the TC gene [1], [3], [4]. Absence of the HC gene in mice, rats, and opossum (Figure 7) correlates with recent findings that mice do not express HC in the protein form, and that TC functionally stands-in for HC in these rodents [13].

In conclusion, we report that only one soluble extracellular cobalamin binder is present in zebrafish (*Danio rerio*). The protein behaves like a structural intermediate between the three known extracellular cobalamin binders, IF, TC, and HC, suggesting the zebrafish protein to be a common ancestor for the cobalamin binders found in higher vertebrates.

## Supporting Information

Table S1 List of TC, HC, and IF-like nucleotide sequences found in registered vertebrates by NCBI and UCSC database search. Databases were searched for the terms “TCN1”, “TCN2”, and “GIF”, and species were listed with database accession numbers.
(PDF)

## References

[1] Morkbak AL, Poulsen SS, Nexo E (2007). Haptocorrin in humans.. Clin Chem Lab Med.

[2] Quardros EV (2010). Advance in the understanding of cobalamin assimilation and metabolism.. Br J Haematol.

[3] Nexo BA, Nexo E (1982). Structural homologies of cobalamin-binding proteins. Tryptic peptide mapping of intrinsic factor, transcobalamin and haptocorrin from man, hog and rabbit.. Biochim Biophys Acta.

[4] Kalra S, Li N, Yammani RR, Seetharam S, Seetharam B (2004). Cobalamin (vitamin B12) binding, phylogeny, and synteny of human transcobalamin.. Arch Biochem Biophys.

[5] Li N, Seetharam S, Lindemans J, Alpers DH, Arwert F etal (1993). Isolation and sequence analysis of variant forms of human transcobalamin II.. Biochim Biophys Acta.

[6] Hewitt JE, Seetharam B, Leykam J, Alpers DH (1990). Isolation and characterization of a cDNA encoding porcine gastric haptocorrin.. Eur J Biochem.

[7] Hewitt JE, Gordon MM, Taggart RT, Mohandas TK, Alpers DH (1991). Human gastric intrinsic factor: characterization of cDNA and genomic clones and localization to human chromosome 11.. Genomics.

[8] Johnston J, Yang-Feng T, Berliner N (1992). Genomic structure and mapping of the chromosomal gene for transcobalamin I (TCN1): comparison to human intrinsic factor.. Genomics.

[9] Li N, Seetharam S, Seetharam B (1995). Genomic structure of human transcobalamin II: comparison to human intrinsic factor and transcobalamin I. Biochem Biophys Res Commun.

[10] NCBI website.. http://www.ncbi.nlm.nih.gov/sites/entrez.

[11] Nexo E, Olesen H (1981). Purification and characterization of rabbit haptocorrin.. Biochim Biophys Acta.

[12] Polak DM, Elliot JM, Haluska M (1979). Vitamin B12 binding protein in bovine serum.. J Dairy Sci.

[13] Hygum K, Lildballe DL, Greibe EH, Morkbak AL, Poulsen SS etal (2011). Mouse transcobalamin has features resembling both human transcobalamin and haptocorrin.. PLoS ONE.

[14] Spence R, Gerlach G, Lawrence C, Smith C (2008). The behaviour and ecology of the zebrafish, Danio rerio.. Biol Rev.

[15] Hansen M, Nexo E (1992). Cobalamin binding proteins in human seminal plasma.. Scand J Clin Lab Invest.

[16] Nexo E, Hansen MR, Konradsen L (1988). Human salivary epidermal growth factor, haptocorrin, and amylase before and after prolonged exercise.. Scand J Clin Lab Invest.

[17] Gottlieb Ches, Lau KS, Wasserman LR, Herbert Vict (1965). Rapid Charcoal Assay for Intrinsic Factor (IF), Gastric Juice Unsaturated B12 Binding Capacity, Antibody to IF, and Serum Unsaturated B12 Binding Capacity.. Blood.

[18] Stupperich E, Nexo E (1991). Effect of the cobalt-N coordination on the cobamide recognition by the human vitamin B12 binding proteins intrinsic factor, transcobalamin and haptocorrin.. Eur J Biochem.

[19] Fieber W, Hoffman B, Schmidt W, Stupperich E, Konrat R etal (2002). Pseudocoenzyme B12 and adenosyl-factor A: Electrochemical synthesis and spectroscopic analysis and two natural B12 coenzymes with predominately base-off constitution. Helv. Chim.. Acta.

[20] Nexo E (1975). A new principle in biospecific affinity chromatography used for purification of cobalamin-binding proteins.. Biochem Biophys Acta.

[21] Fedosov SN, Fedosova NU, Krautler B, Nexo E, Petersen TE (2007). Mechanisms of discriminating between cobalamins and their natural analogues during their binding to the specific transporting proteins.. Biochemistry.

[22] UCSC website, Human genome browser gateway.. http://genome.ucsc.edu/cgi-bin/hgGateway.

[23] http://www.ebi.ac.uk/Tools/msa/clustalw2/.

[24] NCBI BLAST.. http://blast.ncbi.nlm.nih.gov/Blast.cgi.

[25] Fedosov SN, Berglund, Fedosova NU, Nexo E, Petersen TE (2002). Comparative analysis of cobalamin binding kinetics and ligand protection for intrinsic factor, transcobalamin, and haptocorrin.. J Biol Chem.

[26] Wuerges J, Geremia S, Randaccio L (2007). Structural study on ligand specificity of human vitamin B12 transporters.. Biochem J.

[27] Kim S, Carrilo M, Kulkarni V, Jagadeeswaran P (2009). Evolution of primary Hemostasis in Early Vertebrates.. PLoS ONE.

