# Supplementary material for: The Cobalamin-Binding Protein in Zebrafish Is an Intermediate between the Three Cobalamin-Binding Proteins in Human
Source: PLoS One. 2012 Apr 20;7(4):e35660. doi: 10.1371/journal.pone.0035660 (PMC3331988; doi:10.1371/journal.pone.0035660)
Supplement: Table S1 — List of TC, HC, and IF-like nucleotide sequences found in registered vertebrates by NCBI and UCSC database search. Databases were searched for the terms “TCN1”, “TCN2”, and “GIF”, and species were listed with database accession numbers. (PDF) [file pone.0035660.s001.pdf]

Table S1:

TC	HC	IF
**Human** (*Homo sapiens*)<br>NCBI Gene ID: 6948	**Human** (*Homo sapiens*)<br>NCBI Gene ID: 6947	**Human** (*Homo sapiens*)<br>NCBI Gene ID: 2694
**Mouse** (*Mus musculus*)<br>NCBI Gene ID: 21452		**Mouse** (*Mus musculus*)<br>NCBI Gene ID: 14603
**Rat** (*Rattus norvegicus*)<br>NCBI Gene ID: 64365		**Rat** (*Rattus norvegicus*)<br>NCBI Gene ID: 29319
**Chimpanzee** *(Pan troglodytes)*<br>NCBI Gene ID: 470178	**Chimpanzee** *(Pan troglodytes)*<br>NCBI Gene ID: 736117	**Chimpanzee** *(Pan troglodytes)*<br>NCBI Gene ID: 451215
**Chicken** (*Gallus gallus)*<br>NCBI Gene ID: 429737		**Chicken** (*Gallus gallus)*<br>NCBI Gene ID: 770547
**Cow** (*Bos taurus)*<br>NCBI Gene ID: 281518	**Cow** (*Bos taurus)*<br>NCBI Gene ID: 616239	**Cow** (*Bos taurus)*<br>NCBI Gene ID: 616252
**Dog** *(Canis lupus familiaris)*<br>NCBI Gene ID: 486355	**Dog** *(Canis lupus familiaris)*<br>NCBI Gene ID: 612538	**Dog** *(Canis lupus familiaris)*<br>NCBI Gene ID: 449477
**Frog** (*Xenopus Tropicalis)*<br>NCBI Gene ID: 496768		**Frog** (*Xenopus Tropicalis)*<br>NCBI Gene ID: 100490215
**Hog** (*Sus scrofa)*<br>NCBI Gene ID: 100152846	**Hog** (*Sus scrofa)*<br>NCBI Gene ID: 396873	**Hog** (*Sus scrofa)*<br>NCBI Gene ID: 100514273
**Gibbon** (*Nomascus leucogenys)*<br>NCBI Gene ID: 100580343	**Gibbon** (*Nomascus leucogenys)*<br>NCBI Gene ID: 100583879	**Gibbon** (*Nomascus leucogenys)*<br>NCBI Gene ID: 100583542
**Orangutan** *(Pongo abelii)*<br>NCBI Gene ID: 100171681	**Orangutan** *(Pongo abelii)*<br>NCBI Gene ID: 100461033	**Orangutan** *(Pongo abelii)*<br>NCBI Gene ID: 100460421
**Rhesus Macaque** (*Macaca mulatta)*<br>NCBI GeneID: 716085	**Rhesus Macaque** (*Macaca mulatta)*<br>NCBI Gene ID: 698583	**Rhesus Macaque** (*Macaca mulatta)*<br>NCBI Gene ID: 698704
**Marmoset** (*Callithrix jacchus)*<br>NCBI Gene ID: 100393113	**Marmoset** (*Callithrix jacchus)*<br>NCBI Gene ID: 100411100	**Marmoset** (*Callithrix jacchus)*<br>NCBI Gene ID: 100401664
**Horse** (*Equus caballus)*<br>NCBI Gene ID: 100063639	**Horse** (*Equus caballus)*<br>NCBI Gene ID: 100058916	**Horse** (*Equus caballus)*<br>NCBI Gene ID: 10060988
**Lizard** (*Anolis carolinensis)*<br>NCBI Gene ID; 100480568, like	**Lizard** (*Anolis carolinensis)*<br>NCBI Gene ID; 100552218, like	**Lizard** (*Anolis carolinensis)*<br>NCBI Gene ID; 100563706, like
**Zebrafish** *(Danio rerio)*<br>GeneID: 407646, like	**Zebrafish** *(Danio rerio)*<br>GeneID: 566714, like	
**Salmon** (*Salmo salar)*<br>NCBI Gene ID: 100195232, like		
**Platypus** (*Ornithorhynchus anatinus)*<br>NCBI Gene ID: 100076384	**Platypus** (*Ornithorhynchus anatinus)*<br>NCBI Gene ID: 100074132, like	**Platypus** (*Ornithorhynchus anatinus)*<br>NCBI Gene ID: 100074169, like
**Rabbit** (*Oryctolagus cuniculus)*<br>NCBI Gene ID: 100358905, like	**Rabbit** (*Oryctolagus cuniculus)*<br>NCBI Gene ID: 100356690	**Rabbit** (*Oryctolagus cuniculus)*<br>NCBI Gene ID: 100356172
**Panda** (*Ailuropoda melanoleuca)*<br>NCBI Gene ID: 100480568, like	**Panda** (*Ailuropoda melanoleuca)*<br>UCSC NM_001062, like	**Panda** (*Ailuropoda melanoleuca)*<br>NCBI Gene ID: 100482297, like
**Opossum** (*Monodelphis domestica)*<br>NCBI Gene ID: 100031126, like		**Opossum** (*Monodelphis domestica)*<br>NCBI Gene ID: 100028165, like
**Sheep** *(Ovis aries)*<br>UCSC NM_015749, like	**Sheep** *(Ovis aries)*<br>UCSC NM_001062, like	**Sheep** *(Ovis aries)*<br>UCSC NM_017162, like
**Guinea pig** (*Cavia porcellus)*<br>UCSC NM_015749, like	**Guinea pig** (*Cavia porcellus)*<br>UCSC NM_001062, like	**Guinea pig** (*Cavia porcellus)*<br>UCSC NM_017162, like
**Cat** *(Felis catus)*<br>UCSC NM_015749, like	**Cat** *(Felis catus)*<br>UCSC NM_001062, like	**Cat** *(Felis catus)*<br>UCSC NM_017162
**Elephant** *(Loxodonta africana)*<br>UCSC NM_015749, like	**Elephant** *(Loxodonta africana)*<br>UCSC NM_001062, like	**Elephant** *(Loxodonta africana)*<br>UCSC NM_017162, like
